# What might it cost to increase soil organic carbon using no-till on U.S. cropland?

**DOI:** 10.1186/s13021-020-00162-3

**Published:** 2020-12-05

**Authors:** Mark Sperow

**Affiliations:** Division of Resource Economics and Management, School of Natural Resources, West Virginia University, 1194 Evansdale Drive, Morgantown, WV 26506-6108 USA

**Keywords:** CO_2_ offset costs, Conservation tillage, Carbon capture costs, IPCC

## Abstract

**Background:**

Existing research provides estimates of the biophysical potential for increasing soil organic carbon (SOC) stock, however additional research is needed to enhance our understanding of the economic potential for agricultural soils to offset or help reduce CO_2_ emissions. This study derives the marginal cost to increase SOC sequestration by combining SOC sequestration potential estimates developed using the Intergovernmental Panel on Climate Change (IPCC) factors with an existing payment scheme that was designed to increase no-till (NT) adoption on U.S. cropland. The marginal costs of increasing SOC is a function of the amount of SOC that could be increased through NT and the expected cost to landowners of changing management to use NT.

**Results:**

The variability in SOC sequestration rates due to different land-use, management histories, climate, and soils, combined with the 48 unique payment rates to adopt NT, yield over 5,000 unique marginal cost values for increasing SOC sequestration. Nearly 95 percent of the biophysical potential SOC sequestration increase on U.S. cropland (2802 Tg CO_2_ from 140.1 Tg CO_2_ year^−1^ for 20 years) could be captured for less than $100 Mg^−1^ CO_2._ An estimated 64 to 93 percent of the biophysical potential could be captured for less than the low and high estimated costs to capture CO_2_ for geologic storage of $36.36 to $86.06 Mg^−1^ CO_2_, respectively.

**Conclusions:**

Decreasing tillage intensity through adoption of no-till agriculture offers a cost-effective way to offset a portion of increasing global CO_2_ emissions. This research demonstrates that increasing SOC stocks through NT adoption can offset CO_2_ emissions at a lower cost than some other options for preventing CO_2_ from entering the atmosphere.

## Background

Global carbon dioxide (CO_2_) emissions are increasing every year [12]. While U.S. CO_2_ emissions declined between 2013 and 2017 from 5523 to 5271 Tg CO_2_ year^−1^ [13], measurements at Mauna Loa indicate that the atmospheric concentration of CO_2_ increased from around 400 to 410 ppm (parts per million) between 2015 and 2019 (NOAA [34]). A suite of strategies is likely required to prevent additional CO_2_ from entering the atmosphere through carbon capture and sequestration (CCS) and to remove CO_2_ from the atmosphere by increased afforestation, soil carbon sequestration, or directly removing CO_2_ from the atmosphere. Increasing soil organic carbon (SOC) stocks helps to reduce or offset CO_2_ emissions and helps to decrease the CO_2_ emissions that increase the atmospheric concentration levels of CO_2_. The ability for terrestrial systems to store or increase SOC varies spatially depending upon soil characteristics, climate, land-use, land-use change, and management. The SOC stock on cropland may be increased through decreased soil disturbance from tillage, removal of highly erodible land from crop production, reduced bare summer fallow, and inclusion of winter cover crops in the crop rotation [5, 8, 25, 26, 37, 38]. The biophysical potential SOC increase on U.S. agricultural land has been estimated using the method and factors developed by the Intergovernmental Panel on Climate Change (IPCC [45, 46]). Potential SOC sequestration increase on U.S. cropland of 233.6 Tg CO_2_ Eq year^−1^ (million metric tons of CO_2_ equivalent emissions; [44] could offset all US-Environmental Protection Agency (EPA) estimated average annual emission increases of 30.2 Tg CO_2_ Eq from all sources from 1990 to 2010 or nearly 43 percent of annual CO_2_ emissions from U.S. agriculture [13] for at least 20 years.

The economic potential to increase SOC stocks depends upon the cost to change management to increase SOC, the change in SOC that can be achieved, the cost of other CO_2_ emission mitigation activities, and the value of less CO_2_ entering the atmosphere. For SOC sequestration on U.S. agricultural land, the economic potential has been estimated using crop enterprise budgets and 2006 IPCC coefficients [43], mathematical programming and SOC sequestration rates using EPIC [30] or IPCC [29, 47], and econometric analysis and average SOC sequestration rates for the central U.S. [3]. Economic analyses of SOC sequestration have also been performed using similar approaches in Mali [11] and Senegal [10], Australia [28], and India [18]. These analyses compared the difference in profit between conventional tillage and no tillage implemented to increase SOC sequestration to determine the cost of the SOC sequestration. A common approach among these analyses was to vary the carbon price ($5 to $200 Mg^−1^ C) to evaluate the economic potential for SOC sequestration.

Increased no-till (NT) accounted for more than 50 percent of the potential increase in SOC sequestration in U.S. agricultural soils [44]. NT also helps to control soil erosion and runoff, increases water infiltration, enhances soil organic matter concentration, enhances biodiversity, may lower fuel costs, and creates other environmental benefits [27]. Despite these benefits, NT adoption by U.S. agriculture has been slow, with short-term NT (less than five continuous years) used on only 24 percent of the 111 Mha (million hectares) included in a study area in the U.S. [50]. Implementing NT may require new or modified equipment, different management skills, new pest problems to address, different herbicide applications, and other factors that may increase perceived risk [24].

To understand the slow adoption rates of NT, Knowler and Bradshaw [24] analyzed nearly 170 characteristics such as financial, education, age, acres planted, rainfall, etc., identified in 31 studies from 23 published articles to determine the factors that influence adoption of conservation agriculture. They found that as the number of studies that analyzed the same characteristics about willingness to adopt conservation tillage increased, the greater the likelihood of mixed results, thus making the issue less rather than more clear. The primary policy implication of their research is that there are no factors that influence conservation tillage adoption that are universally statistically significant, however financial assistance to help offset initial investments and transition costs seems to be effective [24].

Given the expected benefits to soil health from NT management yet its slow adoption, U.S. programs often address ways to reduce tillage intensity [6, 60] and encourage increased NT use through programs that help offset some of the investment and transition costs of adopting NT [55]. The expected payment necessary to encourage tillage that reduces soil disturbance may be used to help develop the marginal cost curves for carbon sequestration attained with NT. Little published data exist that provide the payment amount required to encourage landowners to adopt NT.

Based on crop enterprise budget data from twenty-three states that present a direct comparison, Sperow [43] found only four southern states where profit was higher using conventional tillage (CT) than NT for corn and soybean operations. Since research seems to indicate a profit advantage of NT over CT, yet adoption of long term NT is still limited, landowners may perceive that NT profit is lower and risk higher. In place of enterprise budgets, an alternative approach is to estimate the cost to convert from CT to NT using data from programs that were designed to enhance soil conservation. The USDA-NRCS administers the Environmental Quality Incentives Program (EQIP), a voluntary program that provides technical and financial assistance to agricultural producers for up to ten years for implementing conservation practices [55]. Practice Code/Name “329–Residue and Tillage Management–No-Till/Strip Till/ Direct Seed” was developed to reduce soil erosion, improve soil organic matter content, reduce carbon dioxide losses, reduce energy use, improve plant-available moisture, and enhance wildlife habitat by managing biomass residue on the soil surface year-round [57]. Each U.S. state provides the criteria for ranking applications to the program, eligible practices, payment rates, and other program requirements to participate in the program based on input from agribusiness, producer groups, conservation organizations, and representatives from other state and federal agencies [55].

The objective of this research is to estimate the cost of increasing SOC on cropland when tillage is reduced to NT. The cost of SOC stock changes from increased NT adoption was estimated using EQIP payments. The change to SOC stocks from conversion to NT was estimated using IPCC factors and data for soil carbon under native vegetation. The approach generates different carbon values (prices) across cropland for SOC sequestration rates that vary by climate, soil, management history, and land use history and the EQIP payment that varies by state. Biardeau et al. [4] used a similar approach using COMET-Planner to estimate SOC and EQIP payments to reflect the cost increase SOC stocks through crop rotation and the addition of mulch to management systems. The research presented here provides additional evidence that policies that encourage NT adoption are cost effective.

## Results and discussion

**Fig. 1:**
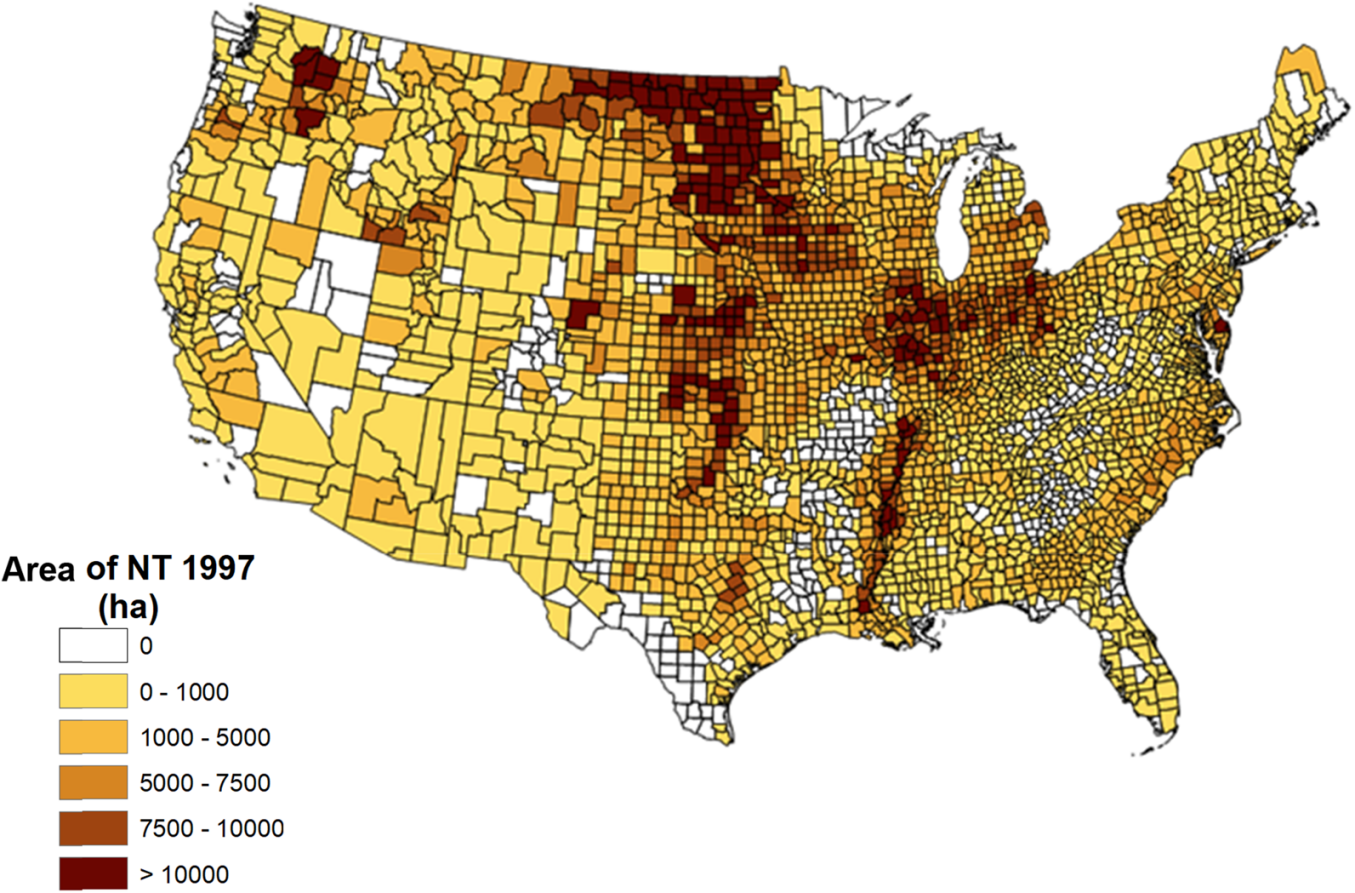
Area of NT (ha) in 1997 based on long term adoption of NT from the Conservation Technology Information Center (CTIC)

The spatial distribution of NT agriculture across the U.S. at the beginning of the analysis in 1997 is from CTIC data (Fig. 1). Based upon the IPCC factors to account for climate, soil characteristics, soil disturbance through tillage, biomass input, and NRI crop data, total SOC stocks increased by NT adoption on U.S. cropland in 2017 was estimated to be 38.2 Tg C year^−1^ (140.1 Tg CO_2_ year^−1^), a rate that could be maintained for 20 years based on the IPCC assumed time to carbon stock equilibrium (Table 1; IPCC [21]). The weighted average SOC sequestration rate by tillage sequence ranged from 0.21 to 0.39 Mg C ha^−1^ year^−1^ (0.8 to 1.4 Mg CO_2_ year^−1^) with no SOC sequestration increase from cropland that was in NT for the final two periods (Table 1). The highest SOC sequestration rate resulted when the management was non-crop in the initial period before transitioning from CT to NT. The smallest SOC sequestration rate resulted from final transitions from reduced tillage (RT) to NT when cropland was CT or non-crop in the initial period.

**Table 1:** Area of U.S. cropland, soil disturbance sequence, total soil organic carbon stored (SOC) annually, total SOC stored after 20 years, and average rate of SOC storage

Tillage Sequence^a^	Area (Mha)	Area (% of total)	Total C (CO_2_) (Tg C year^−1^) (Tg CO_2_ year^−1^)	Total C after 20 years (Tg C year^−1^) (Tg CO_2_ year^−1^)	Total C (% of total)	Weighted average storage rate (Mg C year^−1^) (Mg CO_2_ year^−1^)
CTCTNT	72.3	58.0	27.6 (101.2)	552 (2025)	72.2	0.38 (1.4)
CTRTNT	28.9	23.2	6.2 (22.7)	124 (453)	16.2	0.21 (0.8)
CTNTNT	2.7	2.2	0.0 (0.0)	0	0.0	0.0 (0.0)
RTCTNT	0.1	0.0	0.0 (0.0)	0	0.0	0.33 (1.0)
RTRTNT	1.1	0.9	0.2 (0.7)	5 (18)	0.6	0.23 (0.8)
RTNTNT	6.0	4.8	0.0 (0.0)	0	0.0	0.0 (0.0)
NCCTNT	8.6	6.9	3.3 (12.1)	67 (246)	8.8	0.39 (1.4)
NCRTNT	3.8	3.1	0.8 (2.9)	16 (60)	2.2	0.21 (0.8)
NCNTNT	1.1	0.9	0.0 (0.0)	0	0.0	0.0 (0.0)
Total	124.7		38.2^b^ (140.1)	765 (2803)		

^a^CTCTNT = Conventional tillage (CT) in 1982, CT in 1997 and no-till (NT) in 2017; CTRTNT = CT in 1982, reduced tillage (RT) in 1997 and NT in 2017; CTNTNT = CT in 1982, NT in 1997 and NT in 2017; RTCTNT = RT in 1982, CT in 1997 and NT in 2017; RTRTNT = RT in 1982, RT in 1997 and NT in 2017; RTNTNT = RT in 1982, NT in 1997 and NT in 2017; NCCTNT = Non-cropland (NC, e.g., hay, pasture, etc.) in 1982, CT in 1997 and NT in 2017; NCRTNT = NC in 1982, RT in 1997 and NT in 2017; NCNTNT = NC in 1982, NT in 1997 and NT in 2017

^b^Ref. [44] estimated SOC sequestration from NT adoption of 35.0 Tg C year^-1^ from a smaller area of NT adoption that excluded highly erodible land removed from crop production

**Fig. 2:**
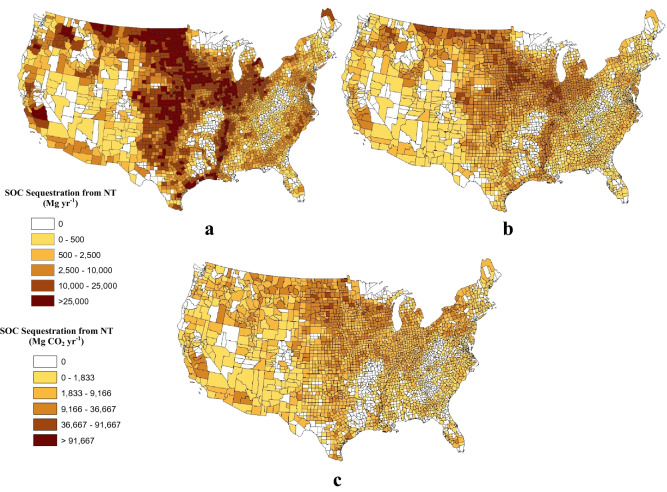
Soil carbon sequestration rates (Mg C year^−1^ or Mg CO_2_ year^−1^) by tillage intensity sequence of CTCTNT (**a**) CTRTNT (**b**), and NCCTNT (**c**)

The tillage sequence CTCTNT provided the highest annual rate of SOC sequestration (27.6 Tg C year^−1^ (101.3 Tg CO_2_ year^−1^) or 72 percent of SOC stock increases of 2803 Tg CO_2_ over 20 years) on the largest area of cropland (72.3 Mha or 58 percent of all cropland) with an overall weighted average of 0.38 Mg C ha^−1^ year^−1^ (1.4 Mg CO_2_ year^−1^; Table 1). The next largest area (29 Mha or 26 percent of cropland) with the tillage sequence of CTRTNT contributed just over 16 percent of the total SOC sequestration potential (453 Tg CO_2_ (22.7 Tg CO_2_ year^−1^ for 20 years)) with an overall weighted average annual rate of 0.21 Mg C ha^−1^ year^−1^ (0.8 Mg CO_2_ year^−1^; Table 1). Combined, these activities accounted for over 88 percent of the increase in SOC stocks from NT adoption on 82.1 percent of the cropland. Agricultural land that was not under a crop (e.g., hay or pasture) in the first inventory, but was under NT in the final inventory following either CT or RT provided an additional 10.9 percent of the SOC sequestration potential on all cropland. Cropland areas with the highest annual SOC sequestration rates when NT was used during the final period with different tillage intensities for the first two periods are presented in Fig. 2. Cropland that transitioned from a lower to higher tillage intensity before NT adoption (e.g., RTCTNT) are not included in Table 1 because SOC increased by only 0.1 Tg CO_2_ year^−1^, with a majority of this increase only at a cost greater than $100 Mg^−1^ CO_2_.

Total SOC sequestration rates provide a broad indication of the biophysical potential for carbon sequestration on cropland. Economic analysis requires information on a smaller scale to help determine the potential costs to increase SOC sequestration, which can help provide an estimate of the potential cost to offset CO_2_ emissions. To provide a better idea of the variability in SOC sequestration rates from NT, the weighted average annual SOC sequestration rates by tillage sequence and Farm Resource Region (USDA-ERS designations that depict geographic specialization in U.S. crop production [52]) are provided in Table 2. SOC sequestration rates ranged from 0.12 Mg C ha^−1^ year^−1^ (0.4 Mg CO_2_ ha^−1^ year^−1^) from the tillage sequence RTRTNT in the Southern Seaboard to 0.47 Mg C ha^−1^ year^−1^ (1.7 Mg CO_2_ ha^−1^ year^−1^) from tillage sequences CTCTNT and NCCTNT in the Northern Crescent Resource Region.

**Table 2:** Weighted average annual soil organic carbon sequestration rates to 30 cm depth by USDA-ERS Farm Resource Region and soil disturbance sequence

Tillage sequence^b^	USDA-ERS Farm Resource Region^a^									Average
	BRR	EUR	FRR	HR	MPR	NCR	NGPR	PGR	SSR	
Mg C ha^−1^ year^−1^										
CTCTNT	0.32	0.41	0.25	0.46	0.44	0.47	0.40	0.32	0.28	0.38
CTRTNT	0.18	0.21	0.16	0.24	0.23	0.24	0.21	0.19	0.15	0.21
CTNTNT	0.00	0.00	0.00	0.00	0.00	0.00	0.00	0.00	0.00	0.00
RTCTNT	0.27	N/A^c^	0.35	0.43	N/A	0.43	0.44	0.13	N/A	0.33
RTRTNT	0.26	0.18	0.13	0.24	0.19	0.24	0.23	0.20	0.12	0.23
RTNTNT	0.00	0.00	0.00	0.00	0.00	0.00	0.00	0.00	0.00	0.00
NCCTNT	0.30	0.41	0.21	0.46	0.38	0.47	0.40	0.33	0.30	0.39
NCRTNT	0.19	0.21	0.15	0.23	0.20	0.24	0.21	0.19	0.16	0.21
NCNTNT	0.00	0.00	0.00	0.00	0.00	0.00	0.00	0.00	0.00	0.00
Overall average	0.25	0.32	0.22	0.34	0.34	0.36	0.31	0.27	0.23	

^a^BRR = Basin and Range Region; EUR = Eastern Uplands Region; FRR = Fruitful Rim Region; HR = Heartland Region; MPR = Mississippi Portal Region; NCR = Northern Crescent Region; NGPR = Northern Great Plains Region; PGR = Prairie Gateway Region; SSR = Southern Seaboard Region

^b^CTCTNT = Conventional tillage (CT) in 1982, CT in 1997 and no-till (NT) in 2017; CTRTNT = CT in 1982, reduced tillage (RT) in 1997 and NT in 2017; CTNTNT = CT in 1982, NT in 1997 and NT in 2017; RTCTNT = RT in 1982, CT in 1997 and NT in 2017; RTRTNT = RT in 1982, RT in 1997 and NT in 2017; RTNTNT = RT in 1982, NT in 1997 and NT in 2017; NCCTNT = Non-cropland (NC, e.g., hay, pasture, etc.) in 1982, CT in 1997 and NT in 2017; NCRTNT = NC in 1982, RT in 1997 and NT in 2017; NCNTNT = NC in 1982, NT in 1997 and NT in 2017

^c^N/A indicates that this tillage sequence was not found in these Farm Resource Regions

While only 48 unique EQIP annual payment rates were used to begin the analysis, when all possible tillage rotation sequences were considered, 5,058 unique carbon prices (cost to increase SOC stocks) that range from a low of $6.36 Mg^−1^ CO_2_ ($23.33 Mg^−1^ C) to $589.33 Mg^−1^ CO_2_ ($2,161 Mg^−1^ C) were generated. The lowest marginal cost occurred when the tillage sequence was CTCTNT in the cold temperate, moist region with a continuous row crop rotation on a high activity mineral soil with an estimated annual SOC sequestration rate of 2.86 Mg CO_2_ ha^−1^ year^−1^. The highest marginal cost occurred on a wheat fallow system in the warm temperate, dry climate region on a sandy soil when the tillage sequence was CTRTNT and the annual rate of SOC sequestration was estimated to be 0.22 Mg CO_2_ ha^−1^ year^−1^ (0.06 Mg C ha^−1^ year^−1^).

**Fig. 3:**
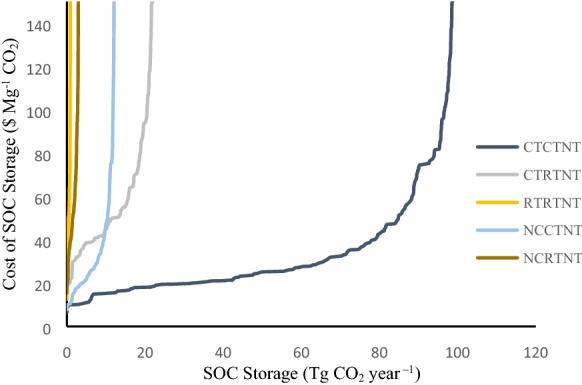
Marginal cost curves to increase soil carbon storage through NT based on EQIP payments for NT adoption based upon the sequence of tillage practices considered. The tillage sequence RTCTNT is not included because the contribution to SOC storage is negligible

**Fig. 4:**
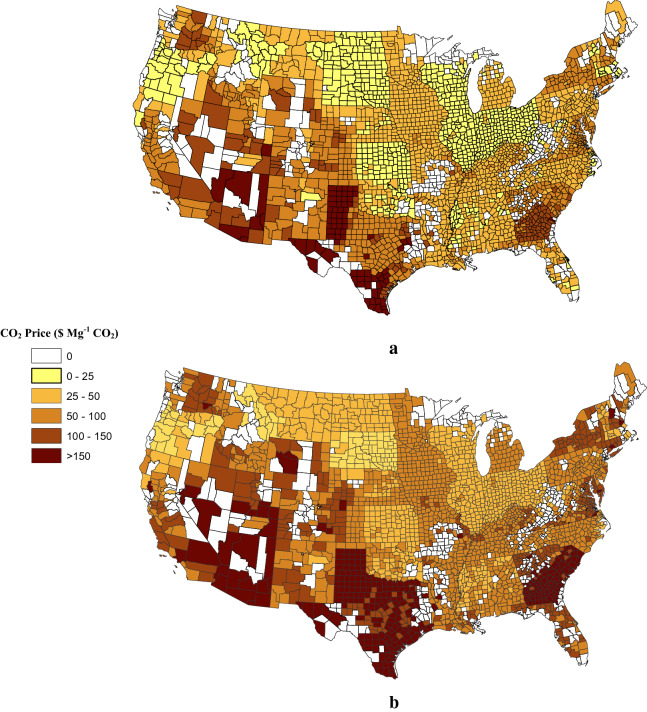
Marginal cost of CO_2_ sequestration by U.S. county for CTCTNT (**a**) and CTRTNT (**b**) tillage

Just over 95 percent or 2660 Tg CO_2_ (133 Tg CO_2_ year^−1^ for 20 years), of the SOC that could be stored through NT adoption could be achieved for less than $100 Mg^−1^ CO_2_ and the marginal costs of achieving 2760 Tg CO_2_ (108.5 Tg CO_2_ year^−1^) or 77 percent of the potential SOC sequestration are less than $50 Mg^−1^ CO_2_. Above 108 Tg CO_2_ year^−1^ the marginal cost of SOC sequestration increased at an increasing rate. The marginal cost curves for increasing SOC stocks in agricultural soils from each tillage sequence are presented in Fig. 3. The least cost regions are predominantly in the midwestern counties, upper Great Plains, and upper northwest for both CTCTNT and CTRTNT tillage sequences (Fig. 4). The results indicate that the most expensive regions for increasing SOC sequestration are predominantly in the southern and southwestern states (Fig. 4) where NT adoption may not be as effective.

**Fig. 5:**
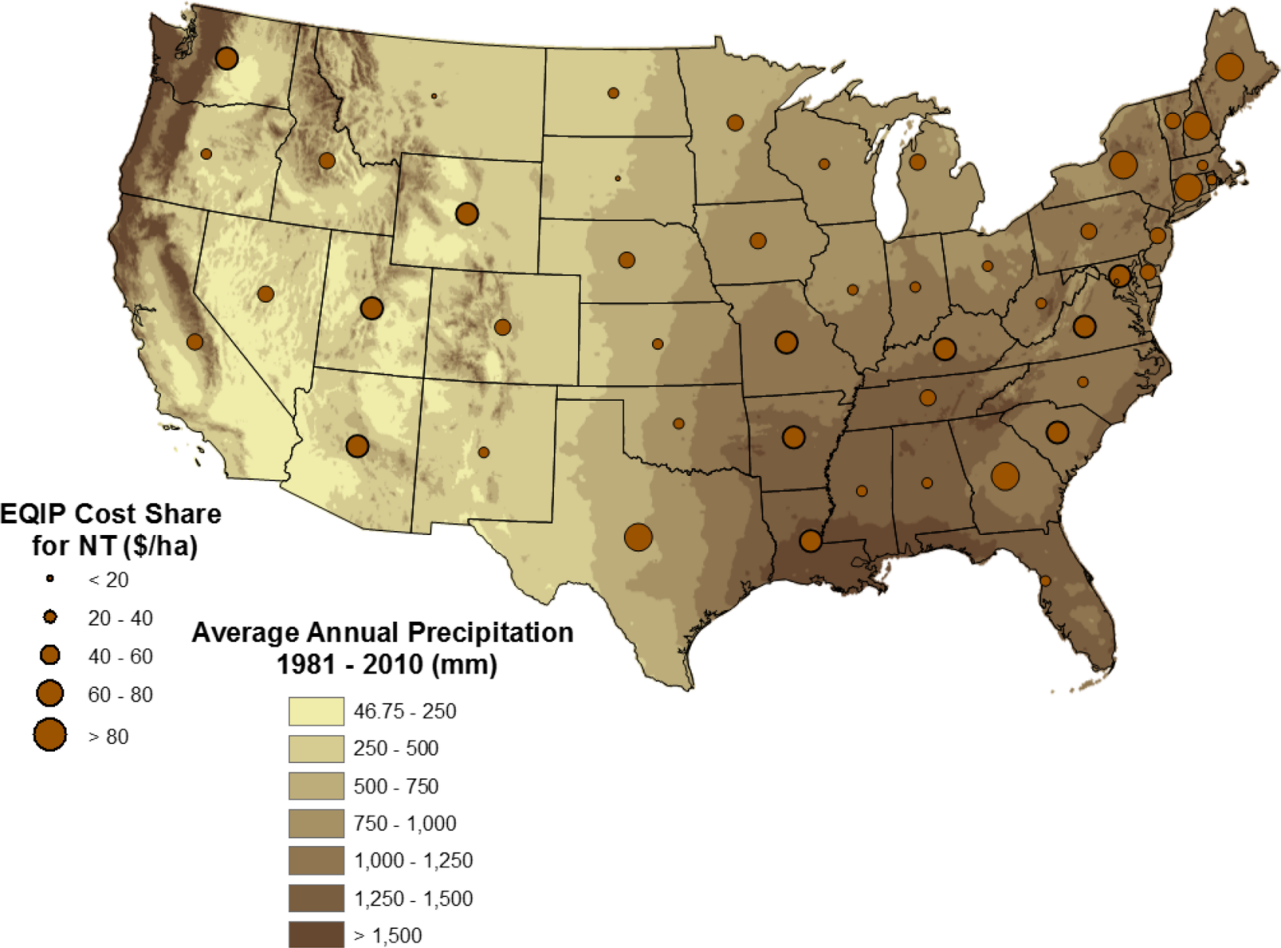
Annual operation costs to convert from a conventional tillage to a no-till system estimated for 2014 ($ ha^−1^) and average rainfall by sate for 1981–2010

Crops respond differently to the amount of soil disturbance during crop production, so the effectiveness of NT depends on many factors [41, 61]. Toliver et al. [49] analyzed 30 years of refereed journal articles documenting 442 comparisons of CT to NT at 92 locations across the U.S. They determined that NT was not as effective on sandy soils where wheat and soybean yields were lower than with CT and that cotton and soybean yields increased with longer time under NT [49]. Rainfall was found to negatively affect crop yields when NT was applied, so NT may increase risk in regions with more annual rainfall. Toliver et al. [49] concluded that the crop, soil characteristics, and climate influence returns, risk, and adoption of NT. The change in average annual precipitation across the U.S., demonstrated with Fig. 5, which shows a gradient that, in general, goes from higher to lower annual moisture from east to west (PRISM Climate Group [39]). The exceptions are the Mississippi Delta region of the south and the far western edge of the Pacific Northwest with the highest annual average precipitation in the country. A comparison of Figs. 1, 2, 3, 4, 5 indicates that, for the most part, NT agriculture is not practiced in regions with high average annual rainfall, consistent with research findings.

The expected costs of NT adoption should be higher in regions with higher precipitation where NT is not as effective. Figure 5 also provides the average cost-share payment for EQIP Practice 329 and precipitation distribution across the conterminous U.S. agricultural land. The highest EQIP payments are in NH, CT, TN, TX, MN, WY, and WA. Of these, only WA contains a high annual precipitation rate and WY was among the states with the lowest precipitation rates. The three lowest annual precipitation rates are all included within TX, which also has the second highest EQIP payment.

Marginal cost curves represent the cost to landowners of adopting NT relative to the amount of SOC that can be increased through the adoption of NT. These marginal cost curves (Fig. 3) demonstrate that there are some landowners that can increase SOC stocks for a lower cost than others. For this analysis, the cost curves represent the minimum CO_2_ price ($ Mg^−1^ CO_2_) required to encourage NT adoption to increase SOC sequestration. This carbon value provides a signal about the cost of one opportunity for addressing CO_2_ emission reductions. Nielsen et al. [33]. estimated that afforestation of cropland could sequester 60 to 130 Tg CO_2_ year^−1^ for $50 to $100 Mg^−1^ respectively, while Biardeau et al. [4] estimated costs of $32 to $442 Mg^−1^ CO_2_ from crop rotation changes and mulch respectively, which is similar to the results presented here. McKinsey and Company [31] provide negative costs for CO_2_ sequestration through tillage reduction globally, indicating that the incentive to adopt NT should be lower than estimated by EQIP and that the profit from crop production under NT is likely larger than CT profit.

Globally, CCS projects that are either under construction or currently operating capture and store about 40 Tg CO_2_ per year [16]. A comparison of the cost to store CO_2_ in geologic formations through CCS to the cost of SOC sequestration provides insights into how the cost to increase SOC sequestration through NT adoption compares to another potential mechanisms to reduce CO_2_ emissions. The estimated CO_2_ storage potential in geologic formations is substantial, over 8300 billion metric tons in saline formations [51], however, little literature is available that provides estimates for the costs to capture, transport, inject, and store CO_2_ from coal fired power plants, a primary source of CO_2_ emissions.

The IPCC [22] estimated cost of CO_2_ capture with geologic storage ranged from $30 to $71 Mg^−1^ CO_2_ (adjusted for this analysis from 2005 to 2014 dollars to be $36.36 to $86.06 Mg^−1^ CO_2_). These estimates are within the range of other cost estimates [2, 9, 15, 20, 32, 40]). The cost of CO_2_ avoidance is considered the average cost to reduce CO_2_ emissions by one unit while providing the same amount of electricity as the reference plant (IPCC [22]).

**Fig. 6:**
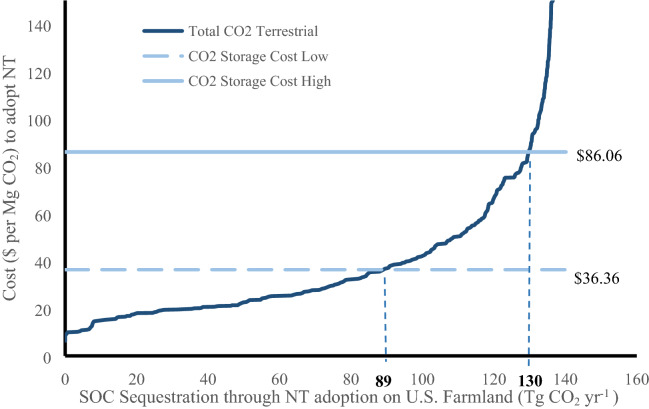
Marginal cost curve for all tillage sequences on cropland that were converted to NT and estimated low and high geologic storage cost ($36.36–$86.06) to capture at a power plant, transport and store CO_2_ in geologic formations (CO_2_ storage cost is converted from $2005 to $2014)

The marginal cost curve for the CO_2_ sequestered through NT adoption is compared to the range of costs estimated for geologic storage of CO_2_ in Fig. 6. Up to 89 Tg CO_2_ year^−1^ could be sequestered through NT adoption at a cost that is less than the lowest estimated average cost for geologic storage. Compared to the higher estimated cost to store CO_2_ in geologic formations, up to 130 Tg CO_2_ year^−1^ could be stored on cropland for a lower cost. These results indicate that terrestrial CO_2_ sequestration on some cropland represents a lower cost alternative to capturing and storing the CO_2_ in geologic formations.

Transitions of historic tillage use to NT influences the effectiveness of NT to increase SOC sequestration. The tillage sequence CTCTNT could capture about 1502 Tg CO_2_ (75.1 Tg CO_2_ year^−1^ for 20 years), which is 84 percent of the potential CO_2_ sequestration in U.S. cropland soil (1780 Tg CO_2_) that could be achieved for less than $36.36 Mg^−1^ CO_2_ over 20 years (Table 3). The additional 9 Tg CO_2_ year^−1^ provided by activities that were not in crops initially but were converted to cropland under CT before moving to NT (tillage sequence NCCTNT) provided enough additional SOC sequestration that the two activities alone could account for 94.4 percent of the potential SOC sequestration on U.S. cropland for a marginal cost lower than the lowest estimated costs for geologic CO_2_ storage. When the high marginal cost of geologic CO_2_ storage was considered, activities that were under the tillage sequence of CTRTNT become the second highest contributor of SOC sequestration by adding 19.1 Tg CO_2_ year^−1^ and, when combined with cropland under tillage sequences CTCTNT and NCCTNT, accounted for over 97 percent of the potential SOC sequestration at that cost. At a cost of less than $86.06 Mg^−1^ CO_2_, these three activities also account for just over 90 percent of the estimated SOC sequestration potential increase on all cropland through NT adoption.

**Table 3:** SOC sequestration to 30 cm depth by tillage sequence relative to expected cost for geologic CO_2_ storage

Upper and lower marginal cost for geologic CO_2_ storage ($Mg^−1^CO_2_)	Tillage sequence^a^ considered in model						Total
	CTCTNT	CTRTNT	RTCTNT	RTRTNT	NCCTNT	NCRTNT	
SOC storage (Tg CO_2_ year^−1^)							
< $36.36	75.1	4.2	0.00	0.1	9.0	0.7	89
< $86.06	95.7	19.1	0.02	0.8	11.7	2.5	130
Proportion of SOC storage for less than the MC							
< $36.36	84.3%	4.7%	0.00%	0.1%	10.1%	0.8%	1
< $86.06	73.7%	14.7%	0.02%	0.6%	9.0%	1.9%	1
Proportion of total annual potential SOC storage (2803 Tg CO_2_)							
< $36.36	53.5%	3.0%	0.00%	0.1%	6.4%	0.5%	63.5%
< $86.06	68.2%	13.6%	0.02%	0.6%	8.4%	1.8%	92.6%

^a^CTCTNT = Conventional tillage (CT) in 1982, CT in 1997 and no-till (NT) in 2017; CTRTNT = CT in 1982, reduced tillage (RT) in 1997 and NT in 2017; CTNTNT = CT in 1982, NT in 1997 and NT in 2017; RTCTNT = RT in 1982, CT in 1997 and NT in 2017; RTRTNT = RT in 1982, RT in 1997 and NT in 2017; RTNTNT = RT in 1982, NT in 1997 and NT in 2017; NCCTNT = Non-cropland (NC, e.g., hay, pasture, etc.) in 1982, NT in 1997 and NT in 2017; NCRTNT = NC in 1982, RT in 1997 and NT in 2017; NCNTNT = NC in 1982, NT in 1997 and NT in 2017

Geologic CO_2_ storage potential is much larger than SOC sequestration increases estimated here, could continue beyond the twenty-years when SOC achieves a new equilibrium, and the risk of CO_2_ release may be lower. It is most likely, however, that the portfolio of options needed to address atmospheric CO_2_ levels will include both, so a comparison of costs provides valuable information.

As described by González-Ramírez et al. [17], SOC sequestration faces the challenges of additionality, ensuring an appropriate baseline, leakage, uncertainty, permanence, monitoring and verification, and distributional effects–such as an impact on crop prices from changes in crop production. Additionality requires that the activities account for increased SOC sequestration that would not have otherwise occurred, or that some landowners would make changes without any payment. In the research presented here, without payment for adopting NT to increase SOC sequestration, about 9.8 Mha or 7.8 percent of cropland was under long term NT (at least five continuous years) in 1997, according to CTIC data. The baseline applied in this research is well defined as the SOC sequestration based on 1997–2017 land use and management on U.S. cropland. Therefore, the increased SOC stocks that are estimated would not have occurred under a business-as-usual scenario and consequently should satisfy the additionality requirement.

The IPCC [21] assesses the uncertainty of SOC estimates based upon the number of studies and variability of study results (Chapter 4; [21]). The uncertainty of land use factors in temperate regions is 9 to 12 percent, under 15 percent for input factors, and under 10 percent for tillage factors [21]. The permanence of SOC sequestration depends on land management. Some of the SOC stock that was increased through NT may be lost if the soil is disturbed through tillage, for example to capture the benefit of increased prices or if yields are reduced, or other reasons. One mechanism to encourage maintaining NT is to retain a portion of the increase in SOC in a reserve pool to serve as insurance against the possibility that the SOC could be released (e.g., the Chicago Climate Change used 20 percent [7]). In addition, measuring and monitoring costs could decrease the payment that could be received by landowners for increasing NT adoption to enhance CO_2_ sequestration. Furthermore, SOC accumulation in agricultural soils is slow and heterogenous across and within individual fields, making short term changes in SOC stocks difficult to measure over time. While remote sensing techniques are continuing to develop that may ease the difficulty of measuring and monitoring, they may still be an issue.

## Conclusion

This study used published estimates for the cost to adopt NT in the U.S. combined with estimates using IPCC factors of the SOC sequestration increases that can be achieved through NT to derive the marginal costs of increasing SOC through these activities. This research demonstrates that SOC sequestration on U.S. cropland through a change in management to NT can efficiently offset a portion of the CO_2_ emissions entering the atmosphere at a cost that is comparable to the costs of geologic CO_2_ storage. The results demonstrate that agricultural soils, while a small sink relative to the large annual emissions from the U.S., could still help mitigate the effects of increased CO_2_ emissions until activities that store substantially more CO_2_ can be developed and implemented.

Estimates indicate that nearly 95 percent of the biophysical potential SOC sequestration increase on U.S. cropland (2803 Tg CO_2_ after 20 years from increases of 140.1 Tg CO_2_ year^−1^) could be captured for a cost less than $100 Mg^−1^ CO_2_ and 64 to 93 percent could be captured for costs less than the lower and higher estimated cost to capture CO_2_ for geologic storage ($36.36 to $86.06 Mg^−1^ CO_2_). Increasing SOC sequestration on U.S. cropland soils could effectively reduce or offset CO_2_ emissions until CO_2_ emissions are drastically reduced or geologic storage costs are reduced through improved technologies, alternative sources of fuel are used for energy, or other technological achievements are implemented.

The value of CO_2_ (price) will ultimately be a function of the instrument used to encourage or enforce CO_2_ emission reductions, the costs of other CO_2_ emission reduction activities, and how much those wishing to reduce emissions would be willing to pay for offsets. Policymakers are still determining whether SOC sequestration could represent a legitimate offset for CO_2_ emission reductions. In the meantime, CO_2_ emissions continue to increase globally.

## Methods

For this analysis, EQIP payments are used to represent the minimum payment required to encourage landowners to adopt NT management. Because EQIP payments capture the costs of implementing multiple conservation practices, not just NT, it should represent a conservative estimate of the cost to adopt NT. The value of increased SOC is then a function of this cost and the amount of SOC increase that results from the decreased soil disturbance. The marginal cost curves that result from NT adoption to increase SOC sequestration on conterminous U.S. cropland were developed for this analysis.

The IPCC developed a method for estimating SOC stock changes to 30 cm based on soil properties, climate, land use, biomass inputs, management activities and other factors, that could be applied using varying levels of detailed input information [23]. The IPCC method was updated with revised factors in 2006 to reflect additional research and improved understanding of SOC sequestration dynamics ([21]; Volume 4, Chapter 5). The land use, tillage, and input factors used to estimate the change in SOC over 20-year inventory periods are fixed and provided in the IPCC literature. The land use factor is determined by the climatic region and how the land was used at the beginning of the inventory (e.g., cropland, set-aside, perennial, etc.). The effect of soil disturbance on SOC is captured by the tillage factor, which varies by the intensity of soil disturbance (CT, RT, and NT), and climate. The input factor accounts for the biomass returned to the soil following crop harvest and varies by the level of input and climatic region [21]. The IPCC approach assumes that SOC achieves equilibrium after 20 years when carbon input is exactly offset by carbon losses, so there is no net change in SOC unless there are additional management or input changes. The IPCC approach and factors have been used to capture the change in SOC that results from transitions of tillage intensity from CT to CT to NT (identified as CTCTNT), from CT to RT to NT (CTRTNT), CT to NT to NT (CTNTNT) etc., with different SOC sequestration rates for each transition and crop rotation [44, 45]. The change in the SOC stock on cropland soils was estimated for this analysis using the 2006 IPCC factors and the approach described by Sperow [44] that traces the transition between crop rotations and tillage intensities through three inventories.

Land use and crop rotations used in this analysis were derived from the 1997 National Resources Inventory (NRI) database which provides data on land use and other activities every 5 years since 1982 [35]. More current data to the same level of detail required for the analysis are not available. Summary NRI data are available for 2007 (USDA-NRCS [59]) and later [54], but these sources do not provide data at the level of detail required for this analysis. Recent NRI reports indicate that there has been little change in the amount of U.S. cropland between 1997 and 2017 (about 2 percent; USDA [53]). Therefore the 20 years of crops grown from the 1982–1997 NRI data applied in the analysis should be adequate for estimating potential SOC sequestration U.S. cropland. The cropping activities addressed in the analysis were derived from the crop rotations established by Eve et al. [14] based upon the predominant crop type (e.g., row crop, small grain, hay, etc.) produced during each 5-year period between NRI surveys (the crop grown in four of the 5 years of data is included in the NRI).

The area of cropland by tillage intensity was derived from data provided by the Conservation Technology Information Center (CTIC) who provided relevant data for tillage intensity by IPCC defined climatic region and crop rotation [14]. Long term (five consecutive years) NT with adequate moisture is necessary in humid and temperate dry climates for NT cropping systems to increase SOC relative to CT [1, 42]. Data for long-term NT adoption provided by the CTIC indicate that, while tillage intensity varies by cropland and climatic region, 1 to 12 percent of U.S. cropland (7.8 Mha total) was under NT in 1997 [14]. A subset of these data show an overall average NT adoption rate of 8.6 percent on the 38 Mha (million hectares) of cropland within the Upper Mississippi River Basin (all or parts of IA, IL, IN, MO, MN, and WI). These data are a little lower than the 13% NT on the smaller area of 4.9 Mha in the Upper Mississippi River Basin [19].

The first inventory addresses the SOC change that results from land use and management activities implemented in 1982. The SOC stock estimates made for 1982 capture the SOC stock at the beginning of the inventory. The first inventory covered from 1982 until 1997. In this analysis, the SOC stock at the beginning of 1997 is the same as the ending stock for the 1982 inventory. If there was no change in land use or management, the SOC stock does not change again and will be zero for the next inventory. Since the IPCC captures the SOC changes between two inventories, the land use and management activities implemented in 1997 were assumed to remain in place until the end of the inventory in 2017.

Potential change in SOC was estimated based on the assumption that the crop rotation in 2017 is the same as the crop rotation and management as at the end of the 1997 inventory. The only change to management was to reduce the level of soil disturbance by adopting NT on all cropland. Land that was already in NT at the end of the 1997 inventory had no additional changes to SOC stocks because there were no additional land use or management changes that alter the SOC stock.

For this analysis, U.S. cropland in 1997 was assessed to determine the potential for increasing the SOC stock through use of NT. All land for which NT adoption was possible was converted to NT for this analysis. Land that was already removed from crop production for the Conservation Reserve Program (CRP) and land that was not managed as cropland (e.g., hay, pasture, etc.) in 1997 were not considered in the analysis because NT could not be applied to them. The NRI data, combined with the IPCC SOC estimation approach, indicate that 124.7 Mha of cropland was used to produce crops in 1997. The CTIC data indicate that there was no cropland managed with NT in 1982, but there was NT cropland in 1997. About 9.8 Mha of cropland was already under NT in 1997, so there was no change in the SOC stock when this cropland continued in NT in 2017. Consequently, only the remaining 114.9 Mha of cropland in the analysis could be converted from a more intensive soil disturbance management system (either CT or RT) to NT.

### Cost to increase NT adoption–EQIP payment data.

EQIP payment data were collected for each U.S. state individually [56, 58] and through the county data provided by USDA-NRCS [57]. EQIP provides for annual payments for up to ten years and NT needs to be maintained for one year for landowners to receive EQIP payments [55]. All of the expected costs of incorporating NT into farm management activities are covered by the EQIP payment, including material, equipment, knowledge accumulation (in some cases), and labor. EQIP payments vary across states but do not vary within a state. For all U.S. states, payments for NT adoption range from $18.29 to $125.73 ha^−1^ (Fig. 5), with an overall average cost share payment of $53.23 ha^−1^.

The highest EQIP payments (greater than $100 ha^−1^) are in the northwest (WA), south (TX) northeast (NH), and the High Plains (WY), each with very different climatic conditions and soil characteristics. It is interesting to note that landowner costs are estimated to be $133.58 ha^−1^ in WA but only $20.95 ha^−1^ in OR, an adjacent state. For landowners in most states, the expected costs of adopting NT vary from $40 to $80 ha^−1^. These costs are higher than estimated by Stonehouse and Bohl [48], yet even though landowner costs would be covered by the EQIP payments, there is still little adoption of NT.

Perceived risk is frequently cited as a reason for the limited adoption of NT in the U.S. [24]. Published EQIP payment schedules indicate that states generally either identify the risk of conservation tillage activities as zero explicitly (LA and TX), or do not include any information about risk (45 states). Colorado is the only state to include an estimate of the increased risk from a change in residue management.

### Estimating marginal costs.

For this analysis, the carbon value is a function of the amount of SOC that can be stored per ha and the state level EQIP payment that was offered to landowners to change from a CT to NT. The EQIP rules for length of payment and requirements for maintaining the practice are not applied because the data are only used to estimate of the cost to change from CT to NT. Most studies present the value of stored carbon in terms of CO_2_. Since SOC is potential CO_2_ in the atmosphere [36], SOC (Mg C ha^−1^ year^−1^) is converted to mass of CO_2_ (Mg CO_2_ ha^−1^ year^−1^) based upon the relative masses (i.e., mass of a CO_2_ molecule to mass of C molecule) by multiplying SOC by 44/12.

The carbon value was estimated as the cost to the landowner of implementing NT (EQIP payment) divided by the carbon sequestration rate (Mg CO_2_ ha^−1^ year^−1^) achieved by adopting no-till using Eq. 1.

1 $$C_{{value}} \; = \;\frac{{EQIP\;Payment\;(\$ \;ha^{{ - 1}} )}}{{MgCO_{2} \;ha^{{ - 1}} \;year^{{ - 1}} }}.$$

## Data Availability

Only summary NRI data are available summary data available at https://www.nrcs.usda.gov/wps/portal/nrcs/main/national/technical/nra/nri/results/, Climate data: http://prism.oregonstate.edu. EQIP data: https://www.nrcs.usda.gov/wps/portal/nrcs/detail/national/programs/financial/eqip/?cid=nrcs143_008223.

## Abbreviations

CT: Conventional tillage
NT: No-tillage
RT: Reduced tillage
CTIC: Conservation technology information center
CTCTNT: Conventional tillage, conventional tillage, no-tillage sequence
CTRTNT: Conventional tillage, reduced tillage, no-tillage sequence
CTNTNT: Conventional tillage, no-tillage, no-tillage sequence
NCCTNT: Non-cropland, conventional tillage, no-tillage sequence
RTCTNT: Reduced tillage, conventional tillage, no-tillage sequence
NC: Non-cropland (grass, pasture, or some other land-use)
EQIP: Environmental quality incentives program
IPCC: Intergovernmental panel on climate change
NRI: National Resources Inventory
SOC: Soil organic carbon
